# The clinical necessity of a distal forearm DEXA scan for predicting distal radius fracture in elderly females: a retrospective case-control study

**DOI:** 10.1186/s12891-023-06265-5

**Published:** 2023-03-09

**Authors:** Sang Beom Ma, Sang Ki Lee, Young Sun An, Woo-suk Kim, Won Sik Choy

**Affiliations:** Department of Orthopedic Surgery, Eulji University College of Medicine, 1306 Dunsan-dong, Seo-gu, Daejeon 35233 South Korea

**Keywords:** Radius fracture, Osteoporotic fractures, Osteoporosis, Dual-energy X-ray absorptiometry, Bone density

## Abstract

**Background:**

Recent studies have demonstrated that the distal forearm dual-energy X-ray absorptiometry (DEXA) scan might be a better method for screening bone mineral density (BMD) and the risk of a distal forearm fracture, compared with a central DEXA scan. Therefore, the purpose of this study was to determine the effectiveness of a distal forearm DEXA scan for predicting the occurrence of a distal radius fracture (DRF) in elderly females who were not initially diagnosed with osteoporosis after a central DEXA scan.

**Methods:**

Among the female patients who visited our institutes and who were over 50 years old and underwent DEXA scans at 3 sites (lumbar spine, proximal femur, and distal forearm), 228 patients with DRF (group 1) and 228 propensity score-matched patients without fractures (group 2) were included in this study. The patients’ general characteristics, BMD, and T-scores were compared. The odds ratios (OR) of each measurement and correlation ratio among BMD values of the different sites were evaluated.

**Results:**

The distal forearm T-score of the elderly females with DRF (group 1) was significantly lower than that of the control group (group 2) (*p* < 0.001 for the one-third radius and ultradistal radius measurements). BMD measured during the distal forearm DEXA scan was a better predictor of DRF risk than BMD measured during the central DEXA (OR = 2.33; *p* = 0.031 for the one-third radius, and OR = 3.98; *p* < 0.001 for the ultradistal radius). The distal one-third radius BMD was correlated with hip BMD, rather than lumbar BMD (*p* < 0.05 in each group).

**Conclusion:**

Performing a distal forearm DEXA scan in addition to a central DEXA scan appears to be clinically significant for detecting the low BMD in the distal radius, which is associated with osteoporotic DRF in elderly females.

**Level of evidence:**

III; case-control study.

## Introduction

As the global population ages, health systems are becoming strained with fragility fractures related to osteoporosis in older adults [1, 2]. Several studies have demonstrated the importance of an early diagnosis of osteoporosis to help prevent these fractures [1, 2]. Osteoporotic fragility fractures usually occur in the distal radius, proximal femur, spine, and proximal humerus, and are associated with socioeconomic costs exceeding $19 billion annually in the United States [1, 3]. Among these, the distal radius fracture (DRF) is the second most common accounting for 37% of overall osteoporotic fractures [4]. DRFs are known to occur approximately 15 years prior to the incidence of a hip fracture, so they have been considered to be a predictor of subsequent osteoporotic fractures [5–8]. A history of DRF in women increased the risk of hip fracture 1.4-fold and the risk of vertebral fracture 5.2-fold compared to women without prior DRF [9]. Therefore, earlier detection of a patient’s DRF risk may prevent further osteoporotic fractures.

Recent studies have demonstrated the necessity of measuring peripheral bone mineral density (BMD) of the distal forearm [10–12]. For this purpose, the distal forearm DEXA scan seems to be a better method for screening BMD and the risk of a distal forearm fracture, compared with a central DEXA scan [3, 10, 12]. The significant association between lower distal forearm BMD and the occurrence of a forearm fracture in the pediatric population has been previously reported [12]. In older adults, lower BMD of the distal forearm was related to an increased risk of osteoporotic fracture in the upper limbs, including the distal radius and proximal humerus. This risk was underestimated when a central DEXA scan was used alone [3, 10]. Additionally, several studies conducted on older adults with DRF showed that BMD measured using a central DEXA scan had no significant correlation with the occurrence of the fracture [13–20]. Moreover, for patients with DRF, there has been a lack of proper evaluation and management for osteoporosis compared with those of vertebral fractures or hip fractures [9, 21–26].

The use of a distal forearm DEXA scan is limited to specific circumstances according to the standard guidelines for the diagnosis of osteoporosis [11, 27]. While the current recommendations are to use the one-third radius of the non-dominant arm to diagnose osteoporosis, there have been some disagreements about the proper sites and methods for measuring distal forearm BMD [10, 11, 28].

Therefore, the purpose of this study was to evaluate the clinical necessity of a distal forearm DEXA scan for predicting DRF in elderly females who were not diagnosed with osteoporosis based on a central DEXA scan.

## Materials and methods

### Study design

We performed a retrospective case-control study using data collected from medical records and radiographic reports between May 2012 and May 2019. The study protocol was reviewed and approved by the institutional review board. All participants provided informed consent before data collection.

**Fig. 1:**
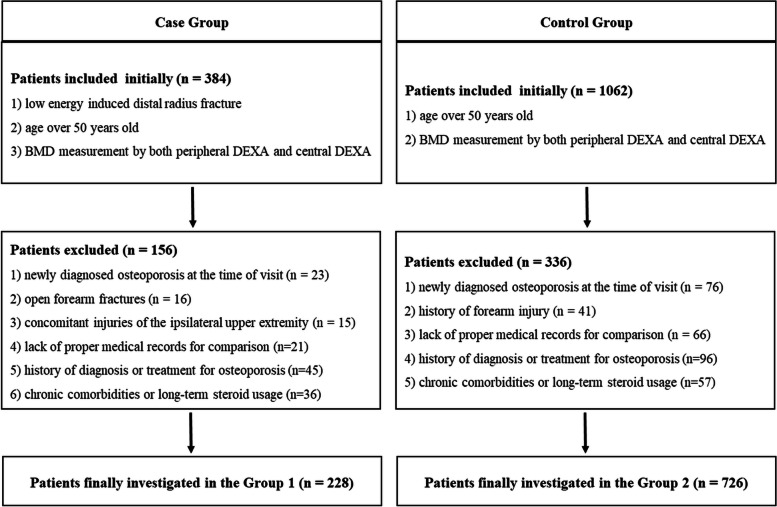
Flow diagram for inclusion and exclusion of the patients in each group

Female patients over 50 years of age who underwent DEXA scans (Lunar Prodigy Advance, GE Healthcare, Madison, WI) at 3 different sites (lumbar spine, proximal femur, and distal forearm) in our hospital were initially classified into two groups. The experimental group consisted of patients with low energy-induced DRF (group 1), and the control group consisted of patients with other injuries excluding fragility fractures (group 2). A detailed list of inclusion and exclusion criteria is described in Fig. 1.

### Bone mineral density measurements

**Fig. 2:**
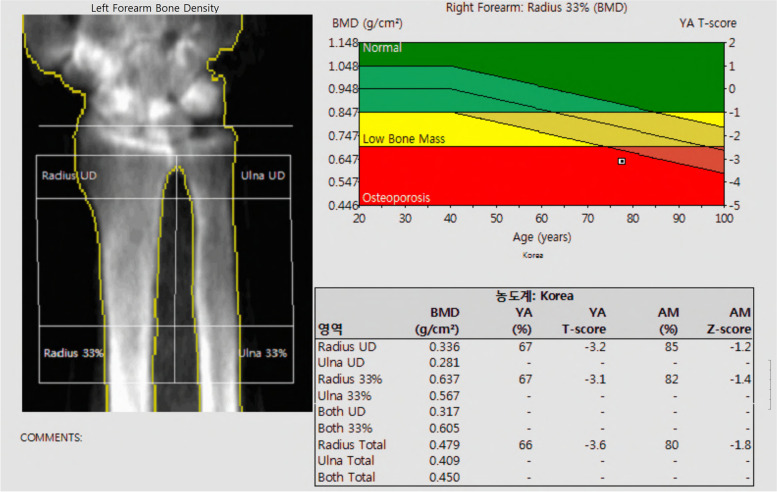
Dual-energy X-ray absorptiometry image of the left forearm. Two regions of interest (ultradistal radius and one-third radius) were included in this study. Radius UD = ultradistal radius, radius 33% = distal one-third radius. The image should include 2 cm of diaphysis over one-third of the forearm and a part of the carpal bones [29]

In this study, we focused on the areal BMD measured using a DEXA scan. For all patients, the central DEXA scan, including the lumbar spine, total hip, and femoral neck, was performed according to the standard guidelines outlined by the International Society for Clinical Densitometry [11, 29]. For spine BMD, the mean values of L2–L4 were used in this study after the exclusion of degenerative and sclerotic lesions and other bone abnormalities. Additionally, we performed a distal forearm DEXA scan at two different sites (ultradistal radius and one-third distal radius) for all patients (Fig. 2). Written informed consent for the additional peripheral DEXA scans were obtained from all study participants. Peripheral BMD was measured using the non-dominant wrist [11] unless the non-dominant wrist was a fracture site, then BMD was measured on the dominant side. This was based on a previous report demonstrating the similarity in BMD between the dominant and non-dominant forearms of healthy patients [30]. The T-score is defined as the number of standard deviations (SD) of a measured BMD value from the average BMD of the reference population (young adult females) [27]. For group 1, the DEXA scan was performed when the fractures were initially diagnosed and the BMD measurements were investigated for this study. For group 2, the BMD values measured when the patients first visited our hospital were used.

### Patient characteristics and clinical evaluations

Patients’ characteristics include age, height, weight, body mass index (BMI), types of fracture according to the AO/OTA classification of a distal radius fracture, Charlson Comorbidity Index (CCI), and history of previous fractures. The Fracture Risk Assessment Tool (FRAX®) was used for assessing the FRAX score indicating the 10-year osteoporosis-related fracture risk [31].

### Statistical analysis

SPSS version 23 (IBM Corporation, Armonk, NY, USA) was used for statistical analyses. Baseline data are reported as mean ± SD, frequencies, or percentages. Independent *t-*test and chi-squared test were used to compare patient characteristics and measurements between groups. Before the comparison, propensity score matching was performed to reduce bias and was calculated for each group based on a logistic regression model using age, height, weight, BMI, CCI, and the side on which they received the distal forearm DEXA scan [32].

We assessed the odds ratio (OR) of each BMD measurement and T-score for all the different sites, and the FRAX score associated with an occurrence of a distal radius fracture using the multivariate logistic regression analysis. In both groups, correlations between BMD measurements for different sites were evaluated using Pearson’s correlation test. The correlation coefficient was interpreted using the following scale: 0.00–0.19, very weak; 0.20–0.39, weak; 0.40–0.59, moderate; 0.60–0.79, strong; and 0.80–1.00, very strong. The paired *t-*test was also used to compare the mean T-score for different sites within each group.

To determine the appropriate number of patients required, we performed a power analysis during the initial study design. We observed that the mean difference in T-score was 0.12 ± 0.35 in our pilot study. The sample size for each group was calculated with 0.05 (two-sided) for α and 0.1 (power = 90%) for β. The analysis identified 186 as the minimum number of subjects required for each group.

## Results

To form group 1, the medical records of 384 female patients with distal radius fractures were initially assessed, and 228 patients were selected to be part of the DRF group (group 1) (Fig. 1). To form group 2, among the 1062 patients initially assessed 726 patients were selected for the control group (group 2). A propensity score was calculated from age, height, weight, BMI, CCI, history of fractures, and the side on which the peripheral DEXA scan was performed (dominant or non-dominant arm). After propensity score matching, 228 patients from group 1 and 228 patients from group 2 were included in the study (Table 1).

**Table 1:** Patients’ demographics

Characteristics	Unmatched cohort			Propensity-matched cohort		
	Group 1 (*n* = 228)	Unmatched group 2 (*n* = 726)	*p*-value	Group 1 (*n* = 228)	Matched group 2 (*n* = 228)	*p*-value
Age (yrs)	60.6 ± 11.2	69.2 ± 10.9	* < 0.001	60.0 ± 10.9	60.6 ± 11.1	0.422
Height (m)	1.54 ± 0.12	1.58 ± 0.11	0.062	1.54 ± 0.12	1.53 ± 0.13	0.473
Weight (kg)	51.9 ± 7.9	50.2 ± 8.1	* 0.021	51.2 ± 7.81	50.9 ± 7.92	0.592
BMI (kg/m^2^)	21.6 ± 3.9	20.2 ± 3.8	* 0.042	21.6 ± 3.9	21.7 ± 4.92	0.700
CCI	0.8 ± 0.7	0.8 ± 0.9	0.231	0.8 ± 0.7	0.8 ± 0.6	0.772
Previous fractures (no. of patients [%])	42 (9.5)	102 (11.5)	* 0.04	42 (9.5)	44 (9.4)	0.234
Peripheral DEXA measured side (no. of patients [%])			–			–
Dominant	217 (49.3)	257 (29.0)	** < 0.001	217 (49.3)	221 (49.4)	0.457
Non-dominant	223 (50.7)	630 (71.0)	** < 0.001	223 (50.7)	226 (50.6)	0.446
Treatment (no. of patients [%])						
Surgery	322 (73)			322 (73)		
Conservative treatment	118 (27)			118 (27)		

The continuous values are given as the mean and the standard deviation

*BMI* body mass index, *CCI* Charlson comorbidity index

* *p* < 0.05; significant difference by Student *t-*test. ** p < 0.05; significant difference by Chi-squared test

A detailed comparison of each measurement between the two groups is described in Table 2.

**Table 2:** Comparison of measurements between the groups

	Group 1	Propensity-matched group 2	***p***-value
T-score			
Femoral neck	−1.62 ± 0.73	− 1.53 ± 0.46	0.081
Total hip	− 1.63 ± 0.72	−1.53 ± 0.51	0.052
Lumbar spine	−1.46 ± 0.89	−1.44 ± 0.65	0.939
One-third radius	−1.89 ± 0.31	−1.56 ± 0.38	* < 0.001
Ultradistal radius	−2.03 ± 0.30	−1.60 ± 0.41	* < 0.001
BMD (g/cm^2^)			
Femoral neck	0.759 ± 0.336	0.774 ± 0.392	0.081
Total hip	0.756 ± 0.232	0.772 ± 0.367	0.052
Lumbar spine	0.966 ± 0.433	0.970 ± 0.351	0.939
One-third radius	0.504 ± 0.214	0.559 ± 0.312	*0.043
Ultradistal radius	0.312 ± 0.094	0.354 ± 0.104	*0.041
FRAX score (%)	5.32 ± 1.17	5.20 ± 1.01	0.251
T-score < −2.5 at distal forearm DEXA (no. of patients [%])	38 (16.7)	17 (7.5)	**0.004

*BMD* bone mineral density, *FRAX* World Health Organization Fracture Risk Algorithm

* *p* < 0.05; significant difference by Student *t*-test. ** *p* < 0.05; significant difference by Chi-squared test

T-scores for the one-third radius and ultradistal radius in group 1 were significantly lower than those in the control group (*p* < 0.001 and *p* < 0.001, respectively). BMD measurements for the one-third radius and ultradistal radius in group 1 were significantly lower (*p* = 0.043 and *p* = 0.041, respectively). Among the patients not initially diagnosed with osteoporosis by a central DEXA scan, the number of patients with a T-score measured at the distal forearm under − 2.5 was remarkably identified in both groups (16.7% of group 1 and 7.5% of group 2), which was also significantly higher in group 1 (*p* = 0.004).

We also assessed the risk factors of the distal radius fracture using a multivariate logistic regression analysis (Table 3). The T-scores calculated for the central DEXA scans did not suggest any correlation with the occurrence of DRF. FRAX score, which was calculated using proximal femur BMD, was not a significant predictor of the occurrence of DRF either. On the contrary, T-scores measured for the one-third distal radius and the ultradistal radius indicated significant risk for DRF (OR = 2.33; *p* = 0.031 and OR = 3.98; *p* < 0.001, respectively).

**Table 3:** Multivariate analysis of risk factors for the occurrence of a distal radius fracture

	OR (95% confidence interval)	***p***-value
**T-score**		
**Femoral neck**	0.26 (0.03, 2.73)	0.263
**Total hip**	0.27 (0.04, 3.12)	0.196
**Lumbar spine**	0.76 (0.52, 1.12)	0.165
**One-third radius**	2.33 (1.08, 5.04)	*0.031
**Ultradistal radius**	3.98 (1.97, 8.01)	* < 0.001
**FRAX score (%)**	0.87 (0.72, 1.04)	0.116

*OR* odds ratio, *BMD* bone mineral density, *FRAX* World Health Organization Fracture Risk Algorithm

* *p* < 0.05; significant difference by multivariate logistic regression analysis

**Fig. 3:**
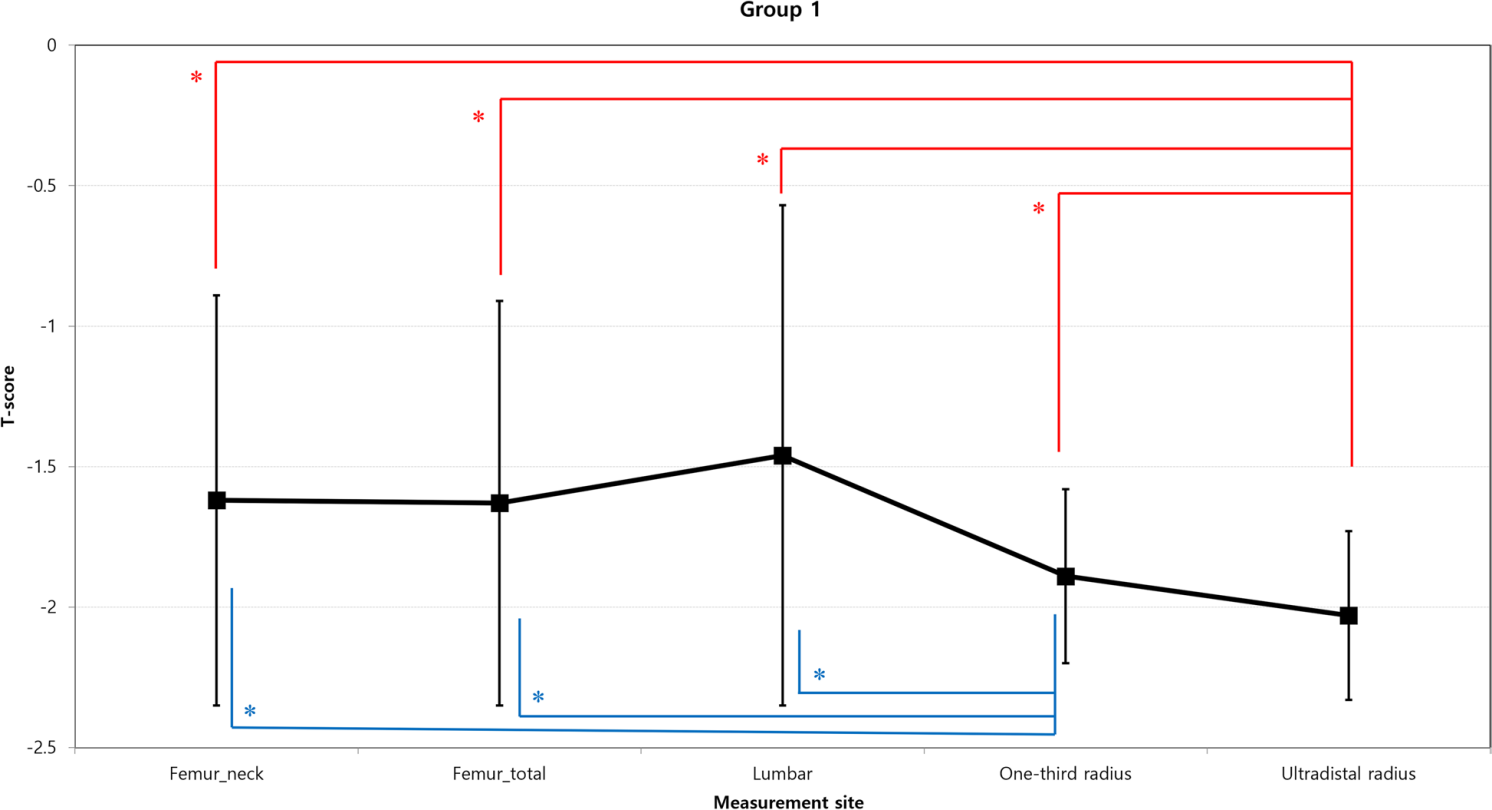
Comparison of mean BMD values measured at each site in group 1. The values are illustrated as the mean (black squares) and standard deviation (bars). * *p* < 0.05; significant difference by the paired *t*-test

**Fig. 4:**
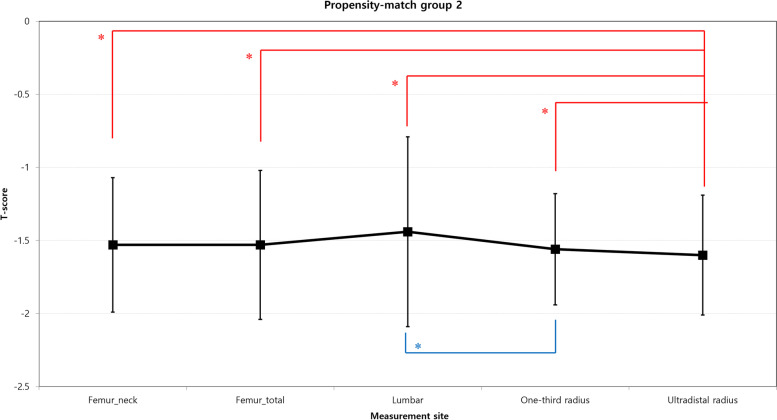
Comparison among the mean BMD values measured at each site in the propensity-matched group 2. The values are illustrated as the mean (black squares) and the standard deviation (bars). * *p* < 0.05; significant difference by the paired *t*-test

The comparisons of mean T-scores measured at each site are shown in Figs. 3 and 4. In group 1, the ultradistal radius T-score was significantly lower than the central and one-third radius DEXA scan T-scores (*p* < 0.05). The one-third radius T-score was also significantly lower than the central DEXA scan T-scores (*p* < 0.05 at each comparison). In group 2, the ultradistal radius T-scores were significantly lower than the central scan T-scores (*p* < 0.05 at each comparison), but were similar to those of the one-third radius. The one-third radius T-score was significantly lower than the lumbar T-score (*p* < 0.05), but was similar to that of the hip.

Correlation coefficients among the BMDs measured at each site are described in Table 4. In group 1, the BMD measured at the femoral neck and the total hip were statistically similar to each other (*p* < 0.001). Lumbar BMD was significantly correlated with BMD of the femoral neck, total hip, and one-third radius (*p* < 0.05 for each analysis). Ultradistal radius BMD measurements showed a significant correlation with the one-third radius BMD (*p* < 0.001). However, it showed no correlation with the other central BMD measurements. On the contrary, one-third radius BMD was significantly correlated with central BMD, which was more strongly correlated with the proximal femur BMD (*p* < 0.05 for lumbar spine BMD and *p* < 0.001 for both proximal femur BMDs). In group 2, proximal femur BMD showed a stronger correlation with the lumbar spine BMD (*p* < 0.001 for both correlations) than with those from group 1. And the one-third radius BMD showed moderate correlation with the proximal femur BMD and the ultradistal radius BMD, similar to that of the group 1(*p* < 0.001 for each correlation).

**Table 4:** Correlation coefficients among the BMD values measured at each site

	Femoral neck	Total hip	Lumbar spine	One-third radius	Ultradistal radius
**Group 1**					
Femoral neck	1	**0.990	*0.296	**0.437	0.031
Total hip		1	*0.203	**0.441	0.037
Lumbar spine			1	*0.132	−0.030
One-third radius				1	**0.624
Ultradistal radius					1
**Propensity-matched group 2**					
Femoral neck	1	**0.980	**0.523	**0.603	0.078
Total hip		1	**0.562	**0.617	0.099
Lumbar spine			1	*0.171	0.043
One-third radius				1 .	**0.645
Ultradistal radius					1

* *p* < 0.05; significant difference by the Pearson correlation test

** *p* < 0.01; significant difference by the Pearson correlation test

## Discussion

There were three main findings in this study. The first is that the T-score of the distal forearm DEXA scan measurements for elderly females with a DRF was significantly lower than the control group. The second is that according to the T-scores, the distal forearm DEXA scan measurements were better predictors of the occurrence of DRF. Finally, the ultradistal radius T-score was the strongest and independent predictor of the DRF. And the distal one-third radius BMD was correlated more closely with the hip BMD than the lumbar BMD. These results suggest that a distal forearm DEXA scan is useful for measuring BMD and better assessing the risk of DRF in patients who were not initially diagnosed with osteoporosis by a central DEXA scan.

The distal forearm T-scores were significantly lower for the fracture group compared with the control group. This indicates that the lower distal forearm DEXA scan measurements may be more closely correlated with the occurrence of a distal radius fracture, especially in patients that might not necessarily be candidates for conventional osteoporosis treatment plans. Our findings that the distal radius BMD measurements were overall lower than the central BMD measurements and that the ultradistal radius BMD measurement was lower than the one-third radius BMD measurement align with what has been reported in existing literature [3, 10, 33]. Moreover, a remarkable number of patients with a T-score under − 2.5 for distal forearm BMD in both groups indicates that measuring distal forearm BMD in addition to central BMD might be helpful for the early detection of osteoporosis and the prevention of further osteoporotic fractures. Recent studies similar to ours also describe the significance of identifying microstructural deficits and low BMD of the distal radius for predicting DRFs or fragility fractures at other sites [3, 10, 12, 34, 35].

We also identified the clinical significance of a distal forearm DEXA scan for earlier diagnosis and prevention of DRFs in both sites (ultradistal radius and distal one-third radius). First, the T-scores for the ultradistal radius were the strongest predictor of a DRF. This is one of the most important findings of this study because the conventional approach to measuring peripheral BMD has focused on the distal one-third radius. Although there is no consensus on which site is better for predicting osteoporosis, the distal one-third radius is the current standard of care [11, 27, 28]. Our findings present a new option that may provide more accurate diagnoses for patients and ultimately better prevent fragility fractures. Second, a previous study described that a lower value for both cortical volumetric and areal BMD for the one-third radius was significantly correlated with DRFs in the pediatric population [12]. However, DRFs in older adults predominantly occur in the distal metaphysis, comprised of both rich cortical and trabecular bone, rather than the diaphysis which is predominantly comprised of cortical bone [36]. Age-related bone loss first occurs in the cancellous bone, then secondary bone, followed by a loss in cortical bone and endocortical surface. Distal radial metaphysis seems to be more affected by age-related changes [3, 37], and this might be reflected by the earlier loss of BMD at the ultradistal radius compared with other sites. Likewise, a previous study described premenopausal women with forearm fractures showing deficits only in trabecular bone volume with the normal cortical area, while older postmenopausal women with forearm fractures showed deficits in the trabecular bone volume and cortical porosity [36]. Second, the distal one-third radius BMD is closely related to the hip BMD. This could be explained by a larger portion of cortical bone affecting the hip BMD, similar to the distal one-third of radial diaphysis. However, the lumbar spine BMD seems to contain a relatively large proportion of cancellous bone and this could sometimes be overestimated due to degeneration of vertebra in elderly patients^3^. Based on all these findings, we anticipate that measuring the distal forearm BMD at both sites would be important. However, as the ultradistal radius BMD measurements showed significant differences and independence from the other sites, distal forearm DEXA scans would be valuable only when performed along with a central DEXA. Moreover, as the conventional methods for predicting osteoporotic fractures including the FRAX score were not adjustable for the population that was not diagnosed with osteoporosis, the distal forearm BMD could be a useful tool for specific circumstances like those in our study design (when osteoporosis was not diagnosed at the central DEXA but identification of bone deficits within the distal radius is required).

The present study had some limitations. First, this was a retrospective, single-center case-control study design, which may have a selection bias. Due to the strict inclusion and exclusion criteria, it only represented a specific subset of older female patients, not the average older adults. Second, some variables that could affect the outcomes such as smoking history, nutritional status, and some serum biochemical markers for bone turnover were not investigated. Third, as the outcomes could be affected by patient position or difference in the region of interest by each measurement, some information biases could exist, especially for the lumbar spine BMD as we only used the average value of L2–L4. Finally, although current research trends have focused on more detailed measurements using quantitative computed tomography (QCT), including volumetric BMD, bone geometry, and distinguishment between the cortical and trabecular area, our study was performed mainly on the DEXA. However, the superiority of the QCT over the DEXA for improving the prediction of fragility fractures has not been established [34]. Further, when using the QCT, more specific circumstances and more detailed measurements are required, and its results may have limited reproducibility in a less-equipped healthcare facility. Moreover, the current standard of care for the diagnosis of osteoporosis is still areal BMD measured by DEXA [11, 27].

## Conclusion

A distal forearm DEXA scan performed in addition to a central DEXA may be an effective tool for detecting the osteoporotic conditions of the distal radius, which is associated with an increased risk of osteoporotic DRF. The ultradistal radius T-score was the strongest value for predicting the DRF and the distal one-third radius DEXA measurements were correlated with the hip DEXA measurements.

## Data Availability

The datasets used and/or analyzed during the current study are available from the corresponding author on reasonable request.

## Abbreviations

DEXA: Dual-energy X-ray absorptiometry
BMD: Bone mineral density
DRF: Distal radius fracture
SD: Standard deviations
BMI: Body mass index
CCI: Charlson comorbidity index
FRAX®: The fracture risk assessment tool
OR: Odds ratio
QCT: Quantitative computed tomography
